# The distribution characteristics of strabismus surgery types in a tertiary hospital in the Central Plains region during the COVID-19 epidemic

**DOI:** 10.1186/s12886-024-03327-7

**Published:** 2024-02-14

**Authors:** Lijuan Lang, Kexin Guo, Luxi Zhang, Jiong Zhang, Yujie Liu, Junbo Rong, Limin Xu, Zhigang Li

**Affiliations:** The First Affiliated Hospital of Zhengzhou University, Zhengzhou, Henan Province 450000 China

**Keywords:** COVID-19 pandemic, Strabismus, Strabismus classification, Strabismus surgery, Strabismus surgery type distribution

## Abstract

**Objective:**

This study aimed to analyze the distribution of different types of strabismus surgery in a tertiary hospital in Central China during the three-year period of the COVID-19 pandemic.

**Methods:**

A retrospective analysis was conducted on the clinical data of strabismus patients who underwent surgery and were admitted to the Department of Strabismus and Pediatric Ophthalmology at the First Affiliated Hospital of Zhengzhou University between January 2020 and December 2022.

**Results:**

A total of 3939 strabismus surgery patients were collected, including 1357 in 2020, 1451 in 2021, and 1131 in 2022. The number of surgeries decreased significantly in February 2020, August 2021, and November and December 2022. Patients aged 0–6 years accounted for 37% of the total number of strabismus surgery patientsr. The majority (60%) of all strabismus surgery patients were diagnosed with exotropia, with intermittent exotropia accounting for the highest proportion (53%). There was no statistically significant difference in the proportion of intermittent exotropia and constant exotropia during the three-year period (*χ*^*2*^ = 2.642, *P* = 0.267 and *χ*^*2*^ = 3.012, *P* = 0.221, respectively). Among patients with intermittent exotropia, insufficient convergence type was the most common form of strabismus (accounting for over 70%). Non-accommodative esotropia accounted for more than 50% of all internal strabismus cases.

**Conclusion:**

During the period from 2020 to 2022, the total number of strabismus surgeries in our hospital did not show significant fluctuations, but there was a noticeable decrease in the number of surgeries during months affected by the pandemic. Exotropia accounted for the highest proportion among strabismus surgery patients. Intermittent exotropia was the most common type among patients undergoing surgery for exotropia, and the most prevalent subtype was the insufficient convergence type. The age distribution of patients varied in different months, with a concentration of surgeries for strabismus patients in the 7–12 years old age group during the months of July and August each year.

## Background

Strabismus, a common and prevalent disease in ophthalmology, not only affects the appearance of patients, but also leads to amblyopia, abnormal binocular vision function, and even psychological inferiority. Epidemiological studies in European countries have shown that esotropia is the most common type of strabismus in Europe [1]. Relevant studies have also been conducted in Asian countries, with results showing that exotropia is the most common type in Asian countries [2]. Some regions in China have conducted epidemiological studies on strabismus, with results similar to those in Asian countries [3, 4]. Our hospital is the largest comprehensive hospital in Henan Province, China, with sufficient sources of strabismus and pediatric ophthalmology professional groups. We hope to use statistical analysis of our clinical data to obtain the distribution pattern of various types of strabismus surgery in the Central Plains region of China, in order to provide reference for clinical work.

## Methods

Collect clinical data of strabismus patients who underwent surgery by the strabismus and pediatric ophthalmology professional group of the First Affiliated Hospital of Zhengzhou University from January 2020 to December 2022, and carry out statistical analysis. All patient information is extracted from the medical record system, including patient names, gender, age, diagnosis, etc.

All patients underwent refraction, best-corrected visual acuity, anterior segment, fundus, intraocular pressure, and strabismus specialty examinations, including corneal reflection test, prism and alternate cover test, Krimsky test for 33 cm and 6 m strabismus, eye movement, four-point light, binocular vision assessment, and Titmus stereo chart.

All strabismus patients were admitted for surgical treatment after strict screening of surgical indications in the outpatient department. The admission diagnosis was classified according to the Chinese Strabismus Diagnosis Expert Consensus [5].

Statistical analysis: SPSS 27.0 was used for statistical analysis. Chi-square test was used to compare the differences in the proportion of various types of strabismus over the three years. If *P* < 0.05 in the comparison between the three groups, further pairwise comparisons were conducted.

## Results

**Fig. 1:**
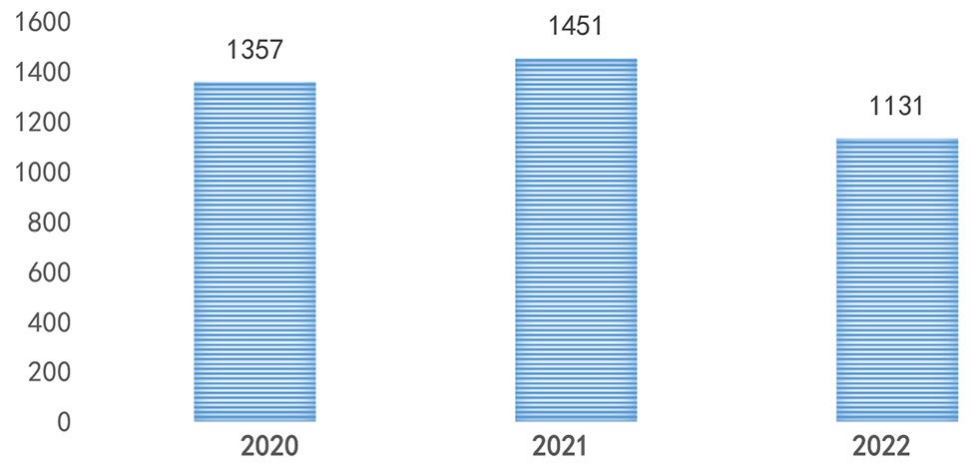
Total number of surgeries per year

**Fig. 2:**
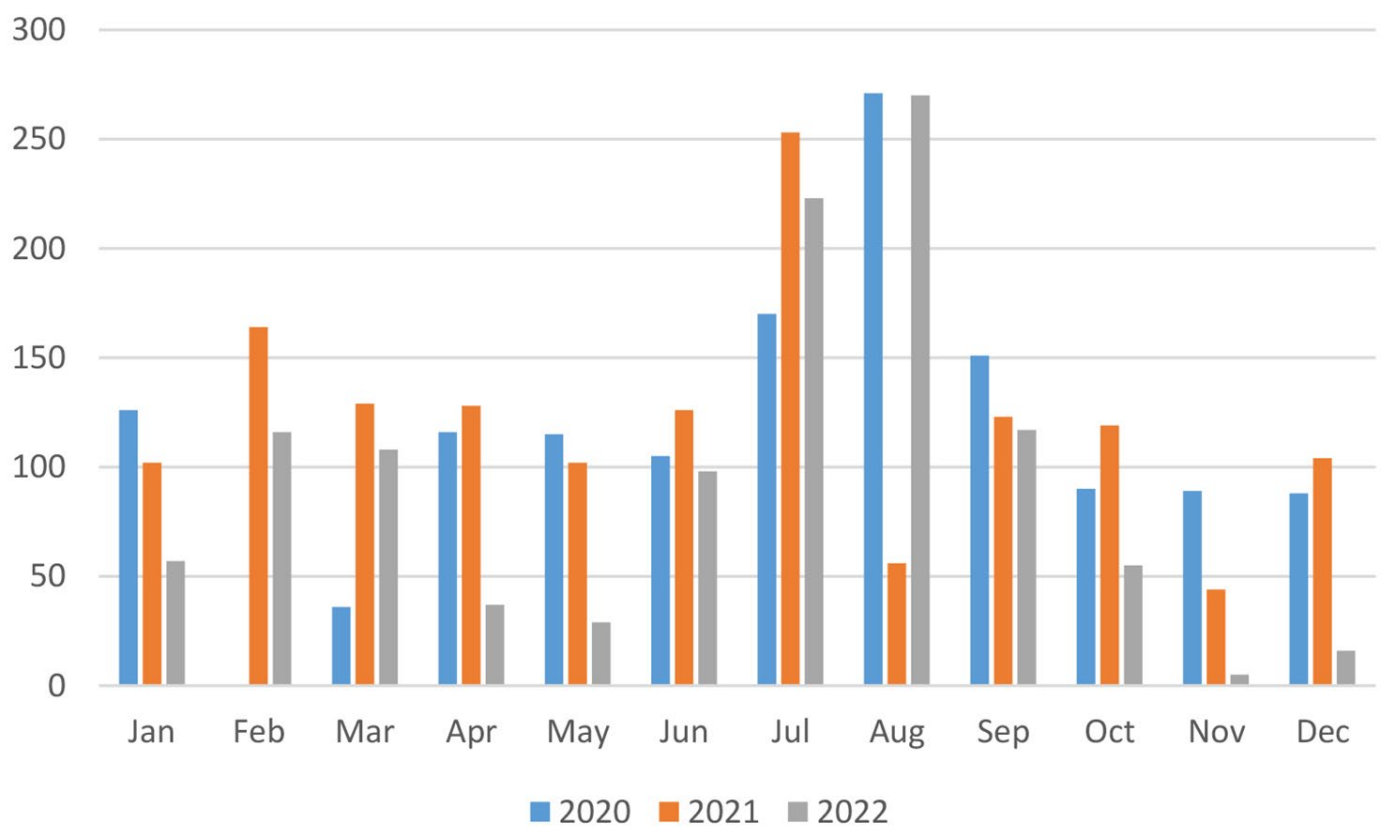
Distribution of total number of strabismus surgeries per month

The total number of strabismus surgeries in 2020 was 1357, in 2021 was 1451, and in 2022 was 1131 (Fig. 1). The surgical volume significantly decreased in February 2020, August 2021, November 2022, and December 2022. Except for special circumstances, July and August are the peak periods for strabismus surgery each year (Table 1; Fig. 2).

**Table 1:** Total number of strabismus surgeries per month from 2020 to 2022

Month Year	Jan	Feb	Mar	Apr	May	Jun	Jul	Aug	Sep	Oct	Nov	Dec	Total
2020	126	0	36	116	115	105	170	271	151	90	89	88	1357
2021	102	164	129	128	102	126	253	56	123	119	44	104	1451
2022	57	116	108	37	29	98	223	270	117	55	5	16	1131

**Fig. 3:**
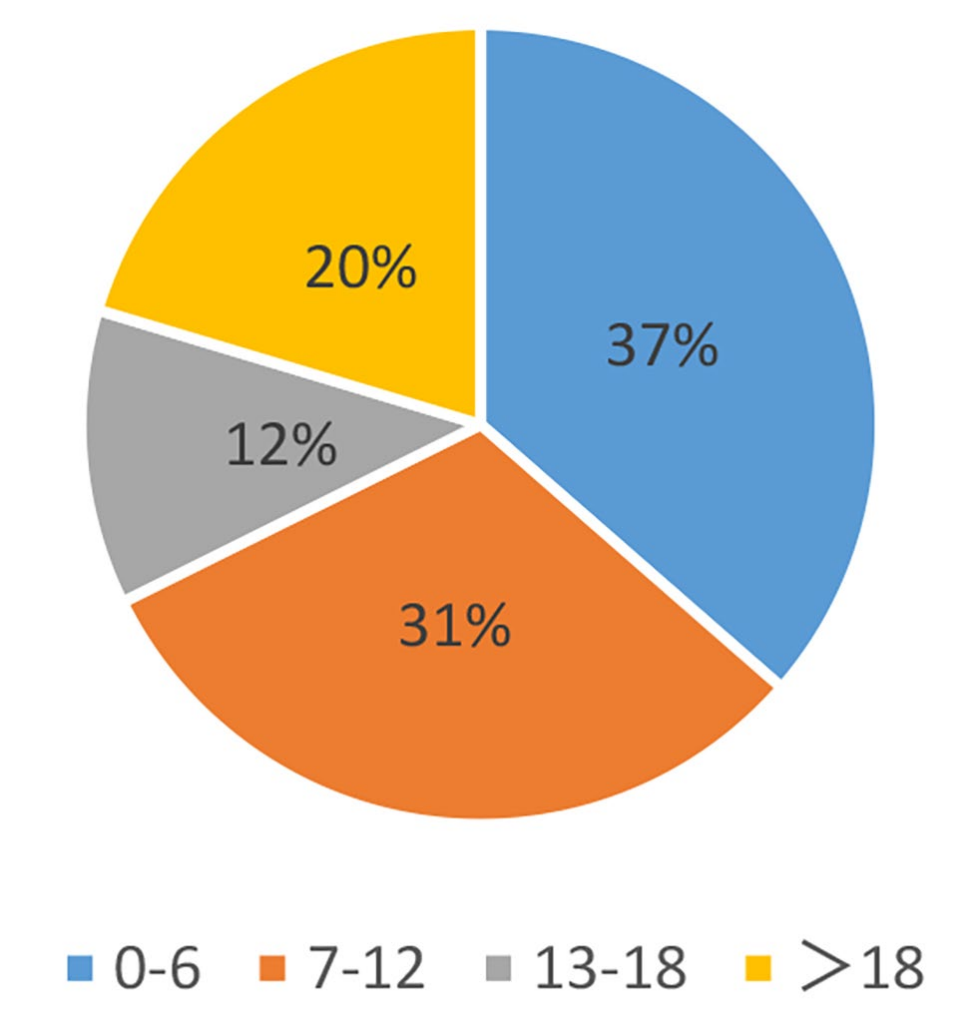
Proportion of total number of strabismus surgeries per year for different age groups

**Fig. 4:**
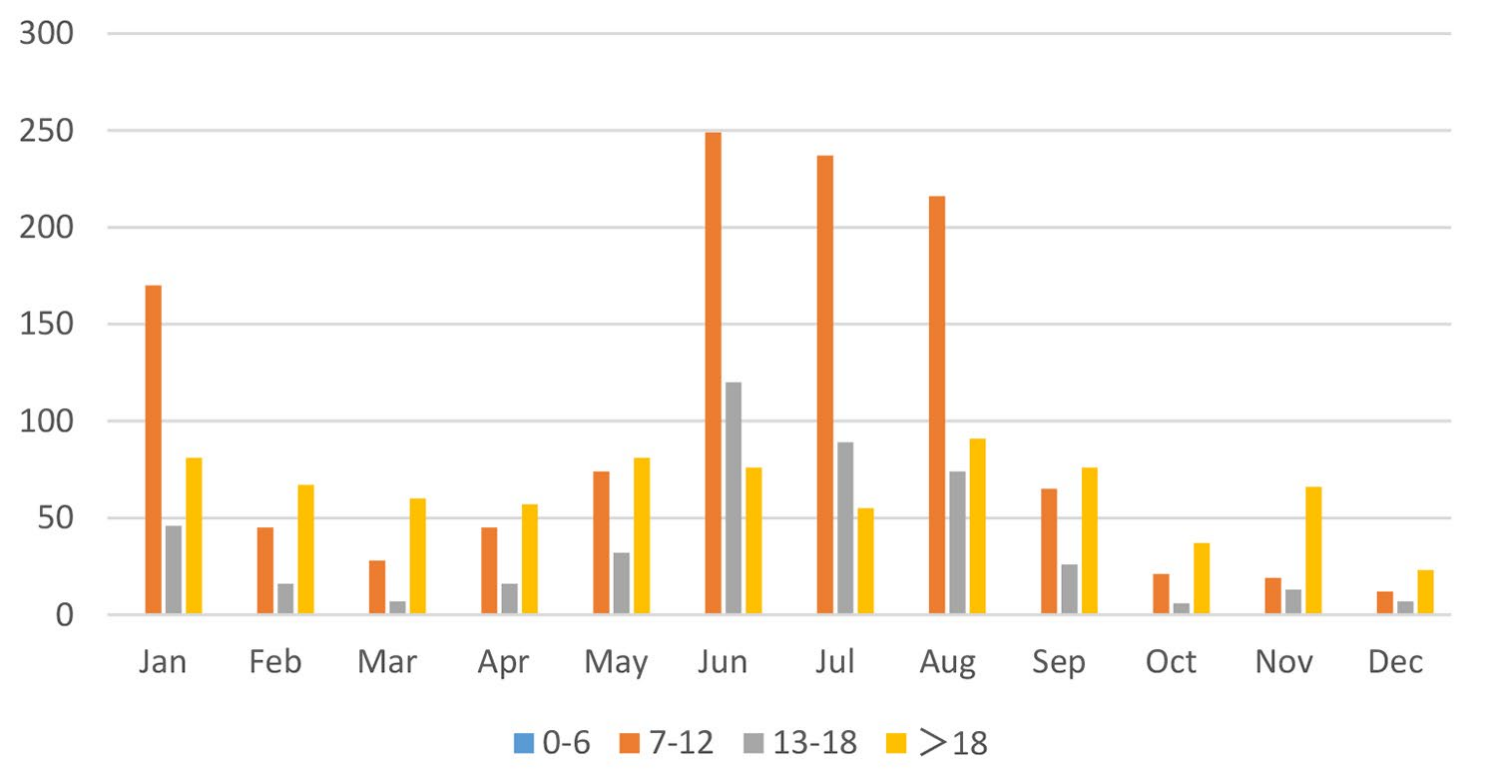
Distribution of total number of strabismus surgeries per month for different age groups

When the patients were grouped by age, it was found that patients aged 0–6 years accounted for 37% of the total number of strabismus surgeries, those aged 7–12 years accounted for 31%, those aged 13–18 years accounted for 12%, and those over 18 years accounted for 20% (Table 2; Fig. 3). It was also found that strabismus surgeries for children aged 7–12 were concentrated in June, July, and August of each year (Fig. 4).

**Table 2:** Distribution of total number of surgeries per month for different age groups

Age(year)	Jan	Feb	Mar	Apr	May	Jun	Jul	Aug	Sep	Oct	Nov	Dec	Total
0–6	109	109	106	129	152	136	115	130	158	75	112	46	1377
7–12	170	45	28	45	74	249	237	216	65	21	19	12	1181
13–18	46	16	7	16	32	120	89	74	26	6	13	7	452
>18	81	67	60	57	81	76	55	91	76	37	66	23	770

From 2020 to 2022, a total of 3939 strabismus surgeries were performed, of which exotropia surgeries were the most common, accounting for 60% (2361 patients); esotropia surgeries accounted for 29% (1146 patients), and the number of exotropia surgeries was about twice that of esotropia surgeries (Table 3). Among exotropia surgeries, intermittent exotropia had the highest proportion, accounting for about 53%, followed by constant exotropia, accounting for about 35%. The proportion of intermittent and constant exotropia did not change significantly over the three years (*χ*^*2*^ = 2.642, *P* = 0.267 and *χ*^*2*^ = 3.012, *P* = 0.221, respectively) (Table 4). Among intermittent exotropia, the proportion of convergence insufficiency type was the highest, accounting for more than 70%, while the proportion of separation excess type was the lowest, less than 3% (Table 5). Among esotropia classifications, non-accommodative esotropia had the highest proportion, accounting for more than 50% (Table 6).

**Table 3:** Distribution of strabismus surgery patients by different types

Year	Exotropia	Esotropia	Vertical and torsional strabismus	Special types of strabismus
2020	830	388	96	43
2021	829	459	97	66
2022	702	299	62	68
Total	2361(60%)	1146(29%)	255(6%)	177(5%)

**Table 4:** Classification of exotropia surgery patients

Year	Intermittent exotropia	Constant exotropia	Sensory exotropia	Secondary exotropia	Non-comitant exotropia
2020	460_a_(55%)	274_a_(33%)	30_a_(4%)	29_a_(4%)	37_a_(4%)
2021	430_a_(52%)	302_a_(36%)	37_a_(5%)	32_a_(4%)	28_a_(3%)
2022	365_a_(52%)	258_a_(37%)	27_a_(4%)	28_a_(4%)	24_a_(3%)
χ^2^	2.642	3.012	0.829	0.285	1.673
*P*	0.267	0.221	0.669	0.874	0.449

Note: Each subscript letter indicates at the 0.05 level, and the column proportions of these categories have no significant differences with each other

**Table 5:** Classification of intermittent exotropia surgery patients

Year	Basic type	Insufficient convergence type	Over-convergence type
2020	129_a_(28%)	324_a_(70%)	7_a_(2%)
2021	117_a_(27%)	309_a,b_(72%)	4_a_(1%)
2022	76_a_(21%)	289_b_(79%)	0_a_(0%)
χ^2^	6.390	8.846	5.446
*P*	0.041	0.012	0.062

Note: Each subscript letter indicates at the 0.05 level, and the column proportions of these categories have no significant differences with each other

**Table 6:** Classification of esotropia surgery patients

Year	Partial accommodative esotropia	Non-accommodative esotropia	Sensory esotropia	Secondary esotropia	Non-comitant esotropia
2020	138(36%)	203(52%)	8(2%)	15(4%)	24(6%)
2021	137(30%)	261(57%)	9(2%)	32(7%)	20(4%)
2022	95(32%)	157(53%)	7(2%)	27(9%)	13(4%)

## Discussion

Over the past three years, the total number of strabismus surgeries performed at our hospital was 1357, 1451, and 1131, respectively, with no significant fluctuations. The surgery volume significantly decreased in February 2020, August 2021, November 2022, and December 2022. Analysis showed that this was due to the severe impact of the COVID-19 epidemic in Zhengzhou during these months. Non-ophthalmic emergency patients did not seek medical attention, resulting in a sharp decline in strabismus surgery during the months with severe epidemics. Foreign studies have also found that during the COVID-19 epidemic, only ophthalmic emergency patients sought medical attention. This is because ophthalmic examinations require face-to-face interaction, which increases the risk of virus transmission, and ophthalmologists are also assigned to care for COVID-19 infected patients [6]. During the epidemic, the total number of strabismus surgeries per year decreased compared to the pre-epidemic period. Before the epidemic, our hospital performed approximately 2000 strabismus surgeries per year, and the epidemic indeed had a significant impact on the total number of surgical patients.

Except for special circumstances, the peak period for strabismus surgery is usually in July and August each year. Statistical analysis of the age distribution of patients showed that strabismus surgery for children aged 7–12 years is concentrated in June, July, and August each year. This is related to the Chinese national conditions, where July and August are summer vacation periods. The majority of strabismus patients are school-age children. To avoid affecting their studies, parents choose longer holidays to allow their children to undergo strabismus surgery. There were no significant differences in this distribution pattern before and after the epidemic. Relevant studies in China have also shown a significant seasonal variation in the number of strabismus patients seeking medical attention, with peak periods during the winter and summer vacations [7, 8].

After grouping the patients by age, it was found that strabismus surgery patients under 18 years old accounted for 80% of the total, indicating that most strabismus patients undergo surgical treatment before adulthood. The purpose of strabismus surgery is not only to improve appearance but also to obtain good binocular visual function. Studies by many scholars in China and abroad have shown that the development of human binocular vision begins in infancy. The sensitive period is from 3 to 5 months after birth, with a peak at 1–3 years old, and development continues until 6–9 years old [9]. Therefore, some scholars suggest that for intermittent or constant exotropia, surgery should be performed before the age of 7 to better restore perceptual function [10]. With the increasing awareness of strabismus and the necessity of strabismus surgery among parents, the window for strabismus surgery has shifted earlier.

In the past three years, exotropia surgery patients accounted for the largest proportion, about 60%, followed by esotropia surgery patients, accounting for about 29%. The number of exotropia surgery patients is about twice as high as that of esotropia surgery patients. Among exotropia surgery patients, the proportion of intermittent exotropia is the highest, about 53%, followed by constant exotropia, accounting for about 35%. Among patients with intermittent exotropia, the proportion of insufficient convergence type is the largest, accounting for over 70%, while the proportion of excessive separation type is the smallest, less than 3%. With the development of China’s economy and health care system, young children can receive early vision screening, such as routine physical examinations in kindergartens. Strabismus patients can be detected early and undergo conservative treatment, such as wearing glasses and vision training. More and more parents can supervise their children to persist in wearing glasses and vision training, which enables some children with accommodative esotropia to restore normal eye position through conservative treatment and avoid surgery. Therefore, exotropia surgery patients are more than esotropia patients, with similar results in many domestic studies. In a retrospective study involving 5,746 strabismus patients, found that exotropia surgery accounted for 63.5% of cases, esotropia surgery accounted for 13.2% of cases, and intermittent exotropia was the most common subtype within exotropia surgery, accounting for approximately 71.3% [11]. However, the primary subtype in intermittent exotropia was different from our study’s findings. In a study of 4,640 strabismus surgery patients, reported that exotropia surgery accounted for 54% of cases, esotropia accounted for 22.1% of cases, and constant exotropia was the most common type within exotropia, although its prevalence decreased over the years [4]. Intermittent exotropia was the next most common type and showed an increasing trend. A study conducted in Singapore on a Chinese population also indicated a ratio of 7:1 for exotropia to esotropia, with the majority of exotropia cases being intermittent (63%). In a study of 12,327 strabismus surgery patients over a 10-year period, found that constant exotropia was the most common type among all subtypes, and the number of exotropia surgery patients was approximately 5.83 times that of esotropia surgery patients [12]. However, a study conducted in a tertiary hospital in Spain over a year and a half period involving 153 patients showed that esotropia accounted for 47.7% of cases, while exotropia accounted for 35.9% [13]. These findings indicate significant differences in the classification of strabismus surgery patients between China and Europe.

During the past three years, the proportion of intermittent exotropia has decreased, and the proportion of constant exotropia has increased, but the difference was not statistically significant (*χ*^*2*^ = 2.642, *P* = 0.267; *χ*^*2*^ = 3.012, *P* = 0.221). This may be because the COVID-19 epidemic has limited the medical treatment of patients with intermittent exotropia, and the condition has gradually progressed to constant exotropia. Some studies have shown that one-third of patients with intermittent exotropia experience a deterioration of their condition after a three-year follow-up [14].

This study conducted a statistical analysis of the distribution of strabismus surgery in our hospital during the three-year period of the Covid-19 pandemic. It identified the characteristics of strabismus surgery distribution during the pandemic period. However, there are certain limitations to this study. It is a retrospective analysis that only reflects the results of a specific period and cannot study the overall incidence rate of strabismus. In the future, it is hoped that research with a larger sample size will be conducted to explore the universality of the distribution characteristics of strabismus and the issue of strabismus prevalence in China.

## Conclusion

During the three-year period of the Covid-19 pandemic, the total number of strabismus surgeries in our hospital did not show significant fluctuations. The number of strabismus surgeries decreased significantly during the months of the Covid-19 pandemic. Patients under 18 years old accounted for 80% of the strabismus surgeries, and patients between 7 and 12 years old were concentrated in the months of July and August each year. Among all strabismus surgery patients, exotropia was the most common type, occurring twice as often as esotropia. Among patients with exotropia, intermittent exotropia had the highest proportion. The combined proportion of intermittent exotropia and constant exotropia remained stable during the three-year period of the Covid-19 pandemic, but the proportion of intermittent exotropia decreased, while the proportion of constant exotropia increased. This fully demonstrates the importance of early screening and regular follow-up observation for patients with intermittent exotropia, and the need for necessary intervention measures at the appropriate time to prevent the progression of intermittent exotropia to constant exotropia.

## Data Availability

The datasets generated and/or analysed during the current study are not publicly available due individual privacy but are available from the corresponding author on reasonable request.
